# Non-invasive Predictors of Human Cortical Bone Mechanical Properties: T_2_-Discriminated ^1^H NMR Compared with High Resolution X-ray

**DOI:** 10.1371/journal.pone.0016359

**Published:** 2011-01-21

**Authors:** R. Adam Horch, Daniel F. Gochberg, Jeffry S. Nyman, Mark D. Does

**Affiliations:** 1 Department of Biomedical Engineering, Vanderbilt University, Nashville, Tennessee, United States of America; 2 Institute of Imaging Science, Vanderbilt University, Nashville, Tennessee, United States of America; 3 Department of Radiology and Radiological Sciences, Vanderbilt University, Nashville, Tennessee, United States of America; 4 VA Tennessee Valley Healthcare System, Nashville, Tennessee, United States of America; 5 Department of Orthopaedics and Rehabilitation, Vanderbilt University Medical Center, Nashville, Tennessee, United States of America; 6 Center for Bone Biology, Vanderbilt University Medical Center, Nashville, Tennessee, United States of America; 7 Department of Electrical Engineering, Vanderbilt University, Nashville, Tennessee, United States of America; McMaster University, Canada

## Abstract

Recent advancements in magnetic resonance imaging (MRI) have enabled clinical imaging of human cortical bone, providing a potentially powerful new means for assessing bone health with molecular-scale sensitivities unavailable to conventional X-ray-based diagnostics. To this end, ^1^H nuclear magnetic resonance (NMR) and high-resolution X-ray signals from human cortical bone samples were correlated with mechanical properties of bone. Results showed that ^1^H NMR signals were better predictors of yield stress, peak stress, and pre-yield toughness than were the X-ray derived signals. These ^1^H NMR signals can, in principle, be extracted from clinical MRI, thus offering the potential for improved clinical assessment of fracture risk.

## Introduction

Current bone diagnostics are incomplete. The estimate of areal bone mineral density (BMD) by dual energy x-ray absorptiometry (DXA) does not fully predict fracture risk: for a given DXA score, there is an unexplained increase in fracture risk with age [1], [2], as well as with progression of various disease states, such as diabetes [3]. The limitations of DXA related to BMD depending on bone size [4] may be somewhat overcome by quantitative computed tomography imaging, but, ultimately, any X-ray based diagnostic is only sensitive to the mineral portion of the bone, which accounts for only ≈43% of bone by volume. The remaining soft-tissue components of bone, including collagen and collagen-bound water, are essentially invisible to DXA and quantitative computed tomography. In contrast, clinical magnetic resonance imaging (MRI), which is based on the ^1^H NMR signal, cannot directly detect bone mineral but is sensitive to the soft tissue of bone. Further, a recent study has demonstrated that ^1^H NMR transverse relaxation time constants (T_2_) distinguishes proton signals from collagen, collagen-bound water, and pore water [5]. With this technology and the idea that the presence and hydration-state of collagen play a critical role in dissipating energy in bone [6], we hypothesized that ^1^H NMR can report on the material strength of bone, and we present here compelling experimental observations of ^1^H NMR, X-ray CT and mechanical tests of cadaveric bone samples which indicate that MRI has the potential to better diagnose fracture risk than DXA.

## Results

**Figure 1:**
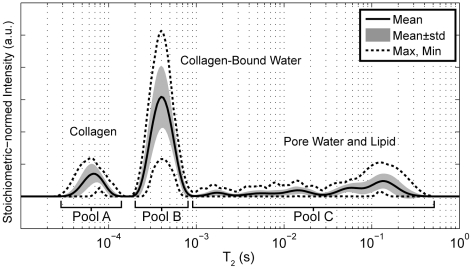
Summary of T_2_ spectra measured from 40 human cortical bone samples. All spectra exhibited a short-T_2_ component (T_2_≈60 µs), derived primarily from collagen protons, an intermediate T_2_ components (T_2_≈400 µs), derived primarily from collagen-bound water protons, and a broad distribution of long-T_2_ components (1 ms<T_2_<1 s), derived from a combination of pore water and lipid protons.

Figure 1 shows the mean (and standard deviation and range) spectrum of ^1^H NMR transverse relaxation time constants (T_2_ spectrum) from 40 cadaveric bone samples. In this mean spectrum and in each individual sample spectrum, signals from three distinct domains of T_2_ were readily identified, as previously found [5]: 50 µs<T_2_<150 µs, defined as pool A, due primarily to collagen methylene protons; 150 µs<T_2_<1 ms, pool B, due primarily to collagen-bound water protons; and 1 ms<T_2_<1 s, pool C, due to water protons in pores in lipid protons. From these three signals, six parameters were extracted: 3 signal amplitudes (*S_A_*, *S_B_*, *S_C_*, in absolutes units of mole ^1^H per liter bone) and 3 corresponding mean relaxation rate constants (*R_2,A_*, *R_2,B_*, *R_2,C_* in s^−1^). Note that while the signal amplitudes are computed in absolute units of concentration, the correspondence between signal amplitudes, *S_A_*, *S_B_*, and *S_C_*, and actual concentrations of collagen methylene protons, bound water protons, and pore-water or lipid protons, respectively, is potentially affected by a number of factors, including the line shape of the methylene protons, the magnetization exchange rate between bound and methylene protons, and overlap of T_2_ components from different sources.

**Figure 2:**
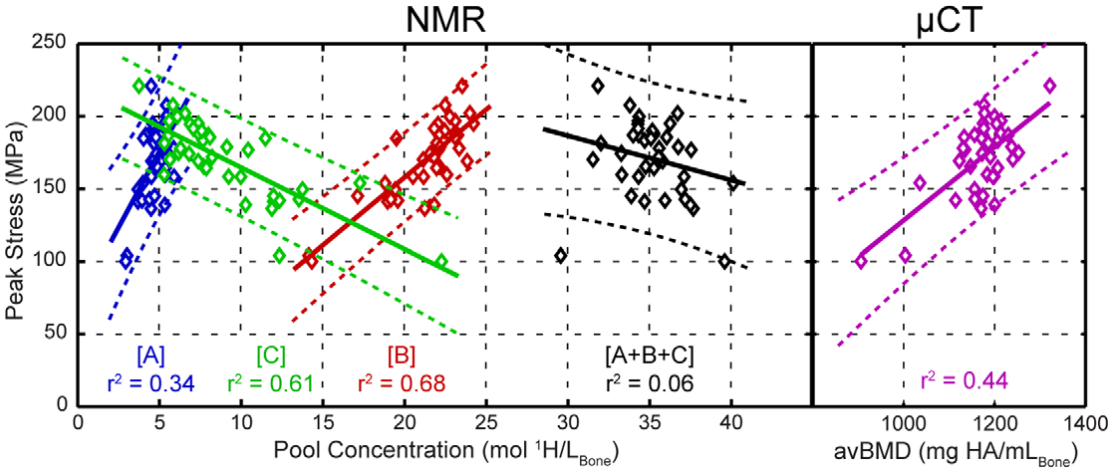
Correlations of measured peak stress and T_2_ spectral component amplitudes (NMR, left) and avBMD measured by μCT (right). Blue, red, and green data show integrated amplitudes (*S_A_*, *S_B_*, and *S_C_*) of the T_2_-discriminated signals from pools A, B, and C, respectively. The black data show the total ^1^H NMR signal (*S_A_*+*S_B_*+*S_C_*), and the purple data are derived from μCT-based measures of avBMD. Each of the NMR signals amplitudes shows a significant linear correlation with peak stress and both *S_B_* and *S_C_* correlate more strongly with peak stress than does avBMD. Note that the total ^1^H NMR signal does not correlate well with peak stress.

Each of the three NMR signal amplitudes (*S_A_*, *S_B_*, *S_C_*) was found to linearly correlate (r^2^ = 0.34, 0.68, 0.61, p<0.05) with peak stress (Fig. 2), but note that the sum of all three signals did not (r^2^ = 0.06, p>0.05). Similar pair-wise linear correlations (and lack thereof) also existed between NMR signal amplitudes and the other three measured mechanical properties. These findings indicate that peak cortical bone stress, and the other measured mechanical properties, are directly related to the amount of collagen and collagen-bound water in bone, and inversely related to the bone pore volume. Micro-computed tomography (μCT)-derived measures of bone porosity and the apparent volumetric bone mineral density (avBMD, akin to DXA) were also found to linearly correlate with mechanical properties, but *S_A_* and *S_B_* were better predictors (i.e., higher r^2^ values) than μCT-porosity for three of four mechanical properties (flexural modulus being the exception), and better predictors than avBMD (i.e., DXA) for all four mechanical properties. Table 1 summarizes the pairwise linear correlations between imaging measure (^1^H NMR and X-ray) and the four mechanical properties.

**Table 1:** A summary of Pearson's r^2^ for pairwise correlations between imaging measures (^1^H NMR and X-ray) and mechanical properties.

	Yield Stress	Peak Stress	Flexural Modulus	Pre-Yield Toughness
*R_2,A_*	0.10	0.12	0.04*	0.12
*R_2,B_*	0.19	0.22	0.12	0.19
*R_2,C_*	0.00*	0.01*	0.01*	0.00*
*S_A_*	0.41	0.34	0.39	0.34
*S_B_*	**0.62**	**0.68**	0.48	**0.57**
*S_C_*	0.57	0.61	0.49	0.49
*S_A_*+*S_B_*+*S_C_*	0.05*	0.06*	0.06*	0.03*
AVBMD	0.43	0.44	0.46	0.33
POROSITY	0.58	0.60	**0.59**	0.46

All correlations were significant (p<0.05) *except* those indicated with ^*^. The imaging measure that was most predictive (highest r^2^) of each mechanical measure is indicated with boldface type.

Note that without the two apparent outlier data (peak stress ≈100 MPa), the predictive power of *S_B_* and *S_C_* decreased to r^2^ values of 0.52 and 0.49, respectively, but the r^2^ of avBMD with peak stress decreased to a greater extent (to 0.16). That is, the relative predictive power of *S_B_* and *S_C_* compared with avBMD *increased* without these two data points. Also note that multiple linear regression analysis told a similar story: combination of NMR signal parameters (*R_B_* and *S_B_*) best predicted of three of four mechanical properties (adjusted R^2^: 0.56-0.70, again, flexural modulus was the exception), and better predicted all four mechanical properties than did avBMD.

## Discussion

As a surrogate to radiation-based CT, MRI has been developed to characterize trabecular volume and architecture as a means to assess fracture risk [7], [8]. For example, such MRI-derived measurements of bone volume fraction and trabecular thickness correlated with the compressive strength of human trabecular bone, although the correlations were not as strong as that between CT-derived BMD and strength [9]. These MRI techniques do not assess the inherent quality of the bone tissue, and this is a significant shortcoming given the importance of ultrastructural characteristics of the extracellular matrix (e.g., collagen integrity) to the fracture resistance of bone [10]. From ex vivo studies of bone, various quantifications of water by NMR have been correlated with the mechanical competence of bone. In a rabbit model of diet-induced hypomineralization, a ^1^H NMR-derived measurement of water content was directly related to the bending strength of cortical bone [11]; however, in a study of ovariectomized and treated mice, only group-mean total water ^1^H NMR signal correlated with mechanical properties—no correlation was found across pooled data from 60 bones, which may be explained by the findings of total ^1^H signal shown here (Fig. 2). Also, an NMR technique known as “decay from diffusion in an internal field” (DDIF) found an inverse correlation between this NMR-derived pore water parameter and the yield stress of bovine trabecular bone in compression [12], in rough agreement with the present observations of pore-water. Prior to the present study though, only one study attempted to correlate NMR measurements of both pore water and water bound to the extracellular matrix to the mechanical properties of human bone [13]. That study used T_2_^*^-discriminated rather than T_2_ -discriminated (used herein) ^1^H NMR signals at low static magnetic field, and while a direct relationship existed between the so-called T_2_^*^-defined bound water and peak stress, it described a much lower fraction of the peak stress variance (r^2^ = 0.36, compared to 0.68, above). Also, the translation of T_2_^*^ based discrimination to clinical imaging may be problematic due to the presence of lipid in bone [5], [11], and the inability of T_2_^*^ to discriminate bone ^1^H pools at clinical field strengths (no discrimination was found at 4.7T [5] and no discrimination has been reported at clinical fields strengths (≥1.5 T)).

**Figure 3:**
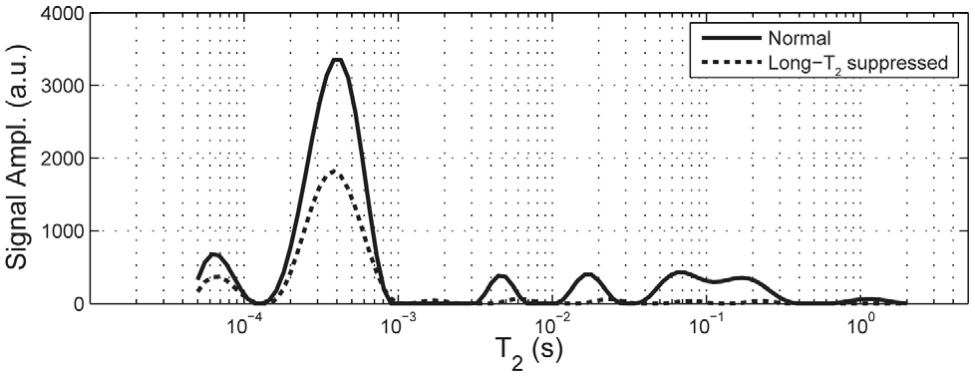
Solid line shows a the T_2_ spectrum from a typical bone sample, and the dotted line shows the spectrum that results following the complex average of two signals, with and without an adiabatic full passage magnetization preparation. The total integrated signal from this long-T_2_ suppressed spectrum is 95% from signals with T_2_<1 ms, thereby demonstrating in principle a simple and practical method for generating a MRI contrast dominated by *S_A_*+*S_B_*.

Current uTE protocols on human MRI systems use echo times <100 µs [14] (and references therein), more than short enough to capture the majority of the bound water signal and some of the collagen proton signal, but the translation of the present findings to clinical MRI will require practical imaging methods of distinguishing these short-T_2_ signals from the longer-T_2_ pore water and lipid signals. There are numerous strategies for integrating T_2_-selective magnetization preparation into a clinically practical uTE-type sequence [15], [16], [17], and the optimal approach for bone imaging has not yet been determined. However, Fig. 3 shows two T_2_ spectra from one bone specimen. The solid line shows the normal T_2_ spectrum, as used in the above analysis, while the dotted line shows the spectrum that results following the complex average of two CPMG signals, with and without the preceding hyperbolic secant radiofrequency (RF) pulse. This RF pulse effectively inverts only the long T_2_ signals while largely saturating the collagen proton and bound-water signal, so the complex average cancels only the long T_2_ signals and results in a net NMR signal that is ≈95% derived from protons with T_2_<1 ms. This result demonstrates in principle that a simple RF pre-pulse, which can be readily integrated into a standard uTE pulse sequence, can distinguish pore water from collagen protons and collagen bound water protons in bone. Once implemented on clinical scanners, such an MRI method can then assess both the contribution of structure to whole bone strength as well as the contributions of collagen integrity and porosity, thus proving a more complete assessment of fracture risk than current X-ray based methods.

## Materials and Methods

### Human cortical bone processing

**Figure 4:**
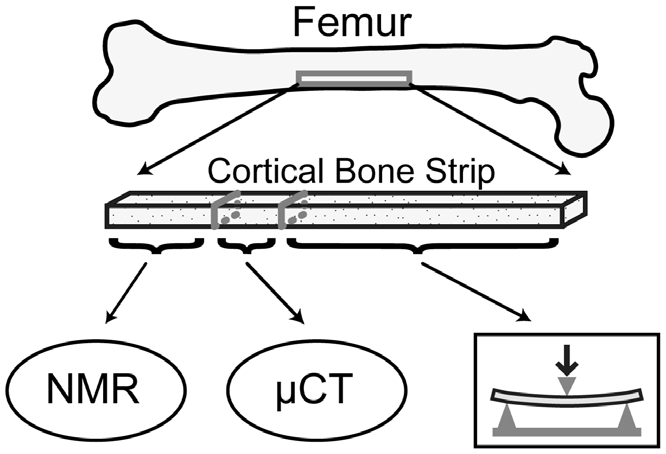
From each cadaveric bone studied, one strip of cortical bone was extracted, three separate pieces of which were used for NMR, μCT, and mechanical testing.

The Musculoskeletal Tissue Foundation (Edison, NJ), a non-profit tissue allograft bank, and the Vanderbilt Donor Program (Nashville, TN) supplied human femurs from 40 cadaveric donors (26 male, 14 female, aged 21-105 years old, mean ± standard deviation: 67±24 years) under instruction to not provide tissue from donors who had tested positive for a blood borne pathogen (e.g., HIV or Hepatitis C). One human cortical bone sample per donor was extracted from the medial quadrant of the mid-shaft and was machined to 70×5×2 mm^3^ dimensions via end mill. During dimensioning, care was taken to remove endosteal and periosteal surfaces such that the final specimens were pure cortical bone. From each milled sample, three specimens were extracted for NMR, μCT, and mechanical testing (Fig. 4). Specimens were stored in phosphate-buffered saline at −20°C then thawed at 4°C for approximately 18 hours prior to NMR measurements. (No more than three freeze-thaw cycles were involved for a given specimen, and separate experiments found that up to six freeze-thaw cycles had negligible impact on the NMR properties.) Final specimen dimensions were measured with digital caliper for volume determination.

### NMR

From one of the three specimens per donor sample, ^1^H NMR transverse relaxation (T_2_) characteristics were measured and reduced to three independent signal components, which we have recently identified as being primarily derived from collagen methylene protons, collagen-bound water protons, and water protons in pores [5]. All NMR measurements were performed in a Varian/Magnex 4.7 T horizontal bore magnet with a Direct Drive Receiver. An in-house loop-gap style RF coil with Teflon structural support was used (similar to the coil described in [18]), which provided 90°/180° RF pulses of ≈8 µs/16 µs duration and contributed negligible background ^1^H signal (≈1% of net HCB signal).

Carr-Purcell-Meiboom-Gill (CPMG) measurements with a total of 10000 echoes were collected at 100 µs echo spacing, which was empirically determined to be a suitable minimum threshold for both maximizing the range of T_2_ detection while minimizing spin-locking effects. Echo magnitudes were fitted to a sum of 128 decaying exponential functions (with time constants log-spaced between 20 µs and 10 sec) in a non-negative least-squares sense, subject to a minimum curvature constraint, which produced a so-called T_2_ spectrum [19]. In order to quantitatively compare the absolute signal amplitudes of T_2_ components across days, a reference sample with long T_2_ (≈2 s) and known proton content was included in each CPMG measurement. The presence of the reference sample, together with the known specimen volumes, enabled the calculation of proton concentrations in the bulk bone specimens for each CPMG relaxation component by comparing integrated areas of each T_2_ spectral component to the area of the marker. As a simple demonstration of the potential for acquiring signal from a specific T_2_ domain without the full CPMG acquisition, from one bone specimen, an additional CPMG measurement was acquired with a preceding a 10-ms duration, 3500 Hz bandwidth hyperbolic secant inversion pulse [20], so chosen to selectively invert the long-T_2_^1^H signal.

### μCT

**Figure 5:**
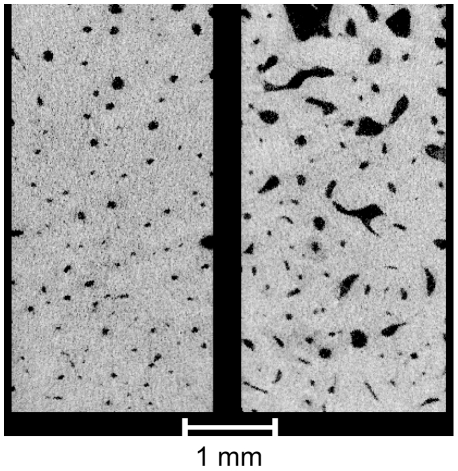
Axial μCT images are shown for cortical bone specimens from a 48 y.o. male donor (left) and an 82 y.o. male donor (right). For the 48 and 82 y.o. donors, respectively, avBMD was 1222 and 1135 mg-HA/cm^3^, and porosity was 4% and 11.3%.

The second specimen from each donor sample (∼ volume of 40 mm^3^) was studied at high resolution (6 µm), with low noise micro-CT (μCT) to quantify apparent volumetric bone mineral density (avBMD) and intracortical porosity (for pores ≥6 µm in diameter). Note that for a given specimen size avBMD is a volumetric analog to areal BMD as measured by DXA, and intracortical porosity at this resolution is not readily determined from clinical radiographs or QCT including high-resolution peripheral QCT scanners (which obtain resolutions of 80–100 µm) [21]. The specimen was scanned by acquiring 1000 projections per 180° at 70 keV using a Scanco, model μCT-40. From an hydroxyapatite (HA) phantom image (acquired weekly), linear attenuation coefficients derived from the μCT images were equated to volumetric bone mineral density (vBMD) in units of mg-HA/cm^3^. Using the Scanco software, the outer perimeter of the sample was defined to determine the total bone volume. The avBMD was defined as the mean of vBMD for all voxels within the total bone volume. The bone tissue volume was segmented from air or soft tissue at a threshold of 800 mg-HA/cm^3^ to determine the porosity ( = 1 minus bone tissue volume per total bone volume) (Fig. 5).

### Mechanical

**Figure 6:**
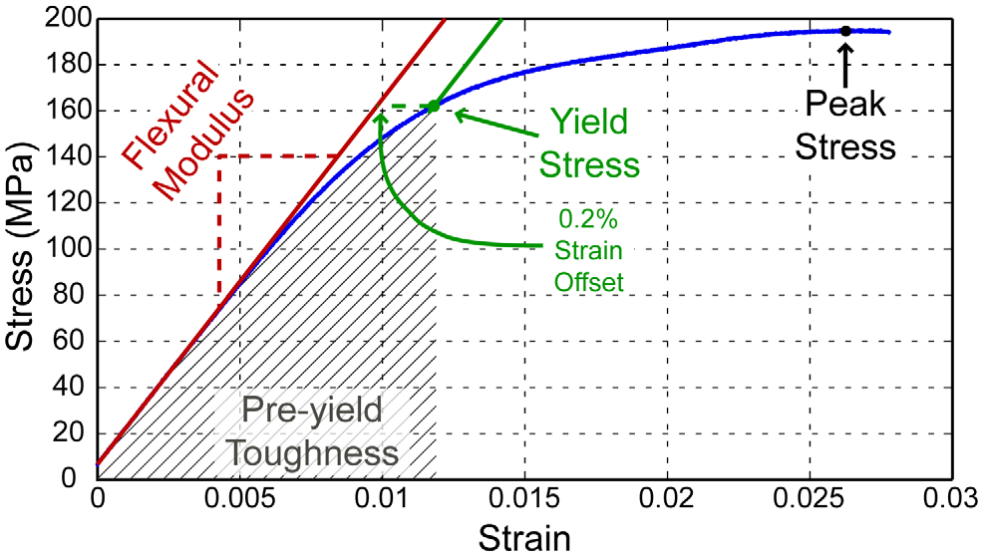
A representative stress vs. strain curve for cortical bone is shown (blue) along with graphical depictions of mechanical parameters. Flexural modulus is the slope of the initial linear mechanical response, yield stress is defined at 0.2% offset from the flexural modulus line, and peak stress is the maximum observed stress. Pre-yield toughness (see text for definition) is proportional to the area under the curve, up to the yield stress.

Finally, we subjected the third, parallelpiped specimen (nominal dimensions of 2 mm×5 mm×40 mm) from each donor sample to a three point bending test, and measured four mechanical properties relevant to fracture risk in bone: yield stress, peak stress, flexural modulus, and pre-yield or elastic toughness. A material testing system (Dynamight 8841, Instron, Canton, OH) recorded the force-displacement data (Fig. 6) from a 100 N load cell and the linear variable differential transformer, respectively, at 50 Hz, as the hydrated bone was loaded to failure at 5 mm/min. The span was 35 mm, and all tests were performed at room temperature. Applying the flexure formula to the yield force, as determined by the 0.2% offset, or to the peak force endured by the bone specimen, and applying the deflection equation to the slope of the linear section of the force-displacement curve provided the material properties, yield stress, peak stress, and flexural modulus, respectively [6]. Pre-yield or elastic toughness was the area under the force-displacement curve from zero displacement to the yield displacement divided by the cross-sectional area of the bone sample to account for slight differences in specimen dimensions.

## References

[1] Kanis JA, Johnell O, Oden A, Dawson A, De Laet C (2001). Ten year probabilities of osteoporotic fractures according to BMD and diagnostic thresholds.. Osteoporos Int.

[2] Johnell O, Kanis JA, Oden A, Johansson H, De Laet C (2005). Predictive value of BMD for hip and other fractures.. J Bone Miner Res.

[3] Vestergaard P (2007). Discrepancies in bone mineral density and fracture risk in patients with type 1 and type 2 diabetes–a meta-analysis.. Osteoporos Int.

[4] Carter DR, Bouxsein ML, Marcus R (1992). New approaches for interpreting projected bone densitometry data.. J Bone Miner Res.

[5] Horch RA, Nyman JS, Gochberg DF, Dortch RD, Does MD (2010). Characterization of 1H NMR Signal in Human Cortical Bone for Magnetic Resonance Imaging.. Magnetic Resonance in Medicine.

[6] Nyman JS, Roy A, Shen XM, Acuna RL, Tyler JH (2006). The influence of water removal on the strength and toughness of cortical bone.. Journal of Biomechanics.

[7] Wehrli FW (2007). Structural and functional assessment of trabecular and cortical bone by micro magnetic resonance imaging.. J Magn Reson Imaging.

[8] Majumdar S (2008). Magnetic resonance imaging for osteoporosis.. Skeletal Radiol.

[9] Majumdar S, Kothari M, Augat P, Newitt DC, Link TM (1998). High-resolution magnetic resonance imaging: three-dimensional trabecular bone architecture and biomechanical properties.. Bone.

[10] Nyman JS, Reyes M, Wang X (2005). Effect of ultrastructural changes on the toughness of bone.. Micron.

[11] Fernandez-Seara MA, Wehrli SL, Takahashi M, Wehrli FW (2004). Water content measured by proton-deuteron exchange NMR predicts bone mineral density and mechanical properties.. J Bone Miner Res.

[12] Sigmund EE, Cho H, Chen P, Byrnes S, Song YQ (2008). Diffusion-based MR methods for bone structure and evolution.. Magn Reson Med.

[13] Nyman JS, Ni Q, Nicolella DP, Wang X (2008). Measurements of mobile and bound water by nuclear magnetic resonance correlate with mechanical properties of bone.. Bone.

[14] Robson MD, Bydder GM (2006). Clinical ultrashort echo time imaging of bone and other connective tissues.. NMR in Biomedicine.

[15] Larson PEZ, Conolly SM, Pauly JM, Nishimura DG (2007). Using adiabatic inversion pulses for long-T-2 suppression in ultrashort echo time (UTE) imaging.. Magnetic Resonance in Medicine.

[16] Rahmer J, Blume U, Bornert P (2007). Selective 3D ultrashort TE imaging: comparison of “dual-echo” acquisition and magnetization preparation for improving short-T-2 contrast.. Magnetic Resonance Materials in Physics Biology and Medicine.

[17] Du J, Takahashi AM, Bae WC, Chung CB, Bydder GM (2010). Dual Inversion Recovery, Ultrashort Echo Time (DIR UTE) Imaging: Creating High Contrast for Short-T-2 Species.. Magnetic Resonance in Medicine.

[18] Horch RA, Wilkens K, Gochberg DF, Does MD (2010). RF coil considerations for short T2 MRI.. Magnetic Resonance in Medicine.

[19] Whittall KP, Mackay AL (1989). Quantitative interpretation of NMR Relaxation Data.. Journal of Magnetic Resonance.

[20] Silver MS, Joseph RI, Hoult DI (1984). Highly Selective Pi/2 and Pi-Pulse Generation.. Journal of Magnetic Resonance.

[21] Burghardt AJ, Kazakia GJ, Ramachandran S, Link TM, Majumdar S (2010). Age- and Gender-Related Differences in the Geometric Properties and Biomechanical Significance of Intracortical Porosity in the Distal Radius and Tibia.. Journal of Bone and Mineral Research.

